# Draft Genome Sequences of 10 Strains of Pseudomonas syringae pv. actinidiae Biovar 1, a Major Kiwifruit Bacterial Canker Pathogen in Japan

**DOI:** 10.1128/MRA.00759-20

**Published:** 2020-08-27

**Authors:** Takashi Fujikawa, Hiroe Hatomi, Hiroyuki Sawada

**Affiliations:** a Institute of Fruit Tree and Tea Science (IFTS), National Agriculture and Food Research Organization (NARO), Tsukuba, Ibaraki, Japan; b Genetic Resources Center (GRC), NARO, Tsukuba, Ibaraki, Japan; University of Arizona

## Abstract

Several groups (biovars) of the kiwifruit bacterial canker pathogen Pseudomonas syringae pv. actinidiae are found in Japan. Here, we sequenced and compared 10 genome sequences of biovar 1, a major group in Japan, which is known as the phaseolotoxin producer.

## ANNOUNCEMENT

The kiwifruit bacterial canker pathogen Pseudomonas syringae pv. actinidiae was first described in Japan in 1989 (1). Subsequently, P. syringae pv. actinidiae was found in other kiwifruit-producing countries (2). Based on comparative analyses (2–4), P. syringae pv. actinidiae was categorized into several groups (biovars). The first Japanese group was named biovar 1 (P. syringae pv. actinidiae biovar 1 [Psa1]), which was also found in Korea in 1989 (2, 5) and in Italy in 1992 (2, 5). This biovar produces phaseolotoxin (2), a phytotoxin that inhibits arginine biosynthesis in host plants and results in bacterial canker symptom development. On the Psa1 chromosome, a large number of genes involved in phaseolotoxin biosynthesis are accumulated in an approximately 23-kb region (*argK-tox* cluster), which is contained in an exogenous genomic island (*tox* island) that Psa1 acquired in the past (2). However, some Psa1 strains found in Ehime Prefecture, Japan (the Ehime isolates), do not produce phaseolotoxin, although they seem to possess the *argK-tox* cluster (6, 7). On the other hand, several Psa1 strains preserved in the NARO genebank (https://www.gene.affrc.go.jp/index_en.php) may lack this cluster (2, 7). Here, we selected 10 strains from the NARO genebank collection (Table 1) that represent Psa1 diversity and conducted comparative genome analyses.

**TABLE 1:** Genome data and accession numbers of strains of Pseudomonas syringae pv. actinidiae biovar 1 and detail for the *argK-tox* gene cluster

Strain	Isolation host, prefecture, yr	Genome information				PGAP^*a*^ annotation			Read information			*argK-tox* gene cluster information^*b*^			
		GenBank accession no.	Genome size (bp)	G+C content (mol %)	No. of contigs (*N*_50_ [bp])	Total no. of genes	No. of rRNAs (5S, 16S, 23S)	No. of tRNAs	SRA^*c*^ accession no.	No. of reads (avg length [bp])	Genome coverage (×)	Phaseolotoxin synthesis^*d*^	*argK-tox* gene cluster	*argK-tox* gene cluster accession no.	Detail
MAFF 302091	Actinidia deliciosa, Kanagawa, 1984	JAAEYK000000000	4,916,203	58.2	2,497 (2,502)	6,374	2, 1, 1	29	SRR11730631	60,196 (236)	7.7	**+**	**+**	MT551019	Same sequence as ICMP 9617
MAFF 302133	Actinidia argute, Kanagawa, 1987	JAAEYI000000000	5,928,911	58.8	653 (14,454)	5,654	3, 1, 1	39	SRR11730639	262,576 (221)	26.4	**+**	**+**	MT551015	Same sequence as ICMP 9617
MAFF 302145	*A. deliciosa*, Wakayama, 1988	JAAEYG000000000	5,164,482	58.4	2,311 (3,012)	6,432	3, 1, 2	28	SRR11730634	97,845 (210)	8.0	**+**	**+**	MT551014	Same sequence as ICMP 9617
MAFF 613024	*A. deliciosa*, Shizuoka, 1995	JAAEYH000000000	4,927,103	58.2	2,470 (2,552)	6,335	2, 1, 1	28	SRR11730633	57,728 (235.6)	7.2	**+**	**+**	MT551013	Same sequence as ICMP 9617
MAFF 211985	*A. deliciosa*, Ehime, 2000	SMHD00000000	5,951,025	58.8	475 (26,121)	5,880	2, 1, 1	44	SRR11730626	286,416 (231.9)	100.1	**+**	**+**	MT551017	Synonymous substitution (silent mutation) in some coding genes against ICMP 9617
MAFF 211981	*A. deliciosa*, Ehime, 2000	JAAEYJ000000000	5,947,905	58.8	524 (19,906)	5,628	3, 1, 1	41	SRR11730636	527,346 (221.5)	42.3	−	**+**	MT551016	Synonymous substitution (silent mutation) in some coding genes against ICMP 9617; frameshift mutation in the fatty acid desaturase gene due to the insertion of a single G
MAFF 211983	*A. deliciosa*, Ehime, 2000	JAAEYF000000000	5,236,580	58.4	2,155 (3,303)	6,342	1, 1, 1	34	SRR11730632	114,619 (230.7)	8.6	−	**+**	MT551018	Synonymous substitution (silent mutation) in some coding genes against ICMP 9617; frameshift mutation in the fatty acid desaturase gene due to the insertion of a single G
MAFF 613017	*A. deliciosa*, Shizuoka, 1986	JAAEYL000000000	5,999,477	58.8	494 (23,006)	5,606	3, 1, 1	44	SRR11730635	813,046 (215.5)	65.0	−	−	−	Absence of *tox* island
MAFF 613018	*A. deliciosa*, Shizuoka, 1986	JAAEYM000000000	5,821,751	58.8	1,046 (9,663)	5,914	1, 1, 1	39	SRR11730637	151,420 (215.5)	16.3	−	−	−	Absence of *tox* island
MAFF 212324	*A. deliciosa*, Shizuoka, unknown	JAAEYN000000000	6,327,049	58.6	609 (17,836)	6,120	2, 1, 1	37	SRR11730638	407,967 (237.2)	34.9	−	−	−	Absence of *tox* island

a PGAP, NCBI Prokaryote Genome Annotation Pipeline.

b +, presence; −, absence.

c SRA, Sequence Read Archive.

d This information comes from Sawada (7).

The strains were cultivated in yeast-peptone (YP) broth at 27°C for 1 day with agitation at 140 rpm. Then, 1-ml aliquots of each culture were used for genomic DNA extraction with a DNeasy minikit (Qiagen, Hilden, Germany). Genomic DNA was sequenced using an Ion Personal Genome Machine (PGM) sequencer with an Ion PGM Hi-Q view OT2 kit (for the library preparation), an Ion PGM Hi-Q view sequencing kit (for the sequencing), and a 318 Chip kit v2 (for the sequencing) (all from Thermo Fisher Scientific, Inc., Waltham, MA, USA). The sequence reads were quality controlled (quality score, <20), and adapter sequences were removed using CLC Genomics Workbench v12 (Qiagen). Using these reads, multiple contigs (filtered with a size longer than 500 bp) were assembled *de novo* using the same software with default parameters (mapping mode = Create simple contig sequences [fast], automatic bubble size = yes, minimum contig length = 500, automatic word size = yes, performing scaffolding = yes, auto-detect paired distances = yes). The draft genomes were annotated using the NCBI Prokaryotic Genome Annotation Pipeline (PGAP) v4. 1 (8).

The guanine and cytosine (G+C) contents and genome sizes for these strains were found to be 58.2% to 58.8% and 4.9 to 6.3 Mbp, respectively (Table 1). PGAP identified 5,606 to 6,432 genes, including multiple rRNA and tRNA genes. In addition, the *argK-tox* cluster of each strain was sequenced by genome walking. Namely, multiple primers were designed with reference to the contig sequences obtained in this study, a large number of amplified fragments of 300 to 600 bp were obtained, and the full sequences of the cluster region were determined by Sanger sequencing. The obtained sequences (Table 1) were compared with the reference genome (GenBank accession no. CM002753) of ICMP 9617 (pathotype strain of P. syringae pv. actinidiae), indicating that some strains have synonymous substitutions (silent mutations) in the cluster. Moreover, in the Ehime isolates (MAFF 211981 and MAFF 211983), it was clarified that a frameshift mutation in the fatty acid desaturase gene occurred due to a single G insertion, possibly resulting in the loss of the ability to produce phaseolotoxin. In the assemblies of MAFF 613017, MAFF 613018, and MAFF 212324, the *tox* island containing the *argK-tox* cluster could not be found, suggesting that the ancestors of these strains may not have experienced the island acquisition event. The fact that such diversification has occurred in the *argK-tox* cluster is an important piece of evidence for elucidating the pathogenicity, ecology, and evolution of Psa1.

### Data availability.

All sequences identified in this study have been deposited in GenBank (see Table 1 for accession numbers).
